# The Transmembrane Domains of TNF-Related Apoptosis-Inducing Ligand (TRAIL) Receptors 1 and 2 Co-Regulate Apoptotic Signaling Capacity

**DOI:** 10.1371/journal.pone.0042526

**Published:** 2012-08-03

**Authors:** Simon Neumann, Tobias Bidon, Marcus Branschädel, Anja Krippner-Heidenreich, Peter Scheurich, Malgorzata Doszczak

**Affiliations:** 1 Institute of Cell Biology and Immunology, University of Stuttgart, Stuttgart, Germany; 2 Institute of Cellular Medicine, Musculoskeletal Research Group, Faculty of Medical Sciences, Newcastle University, Newcastle upon Tyne, United Kingdom; IISER-TVM, India

## Abstract

TNF-related apoptosis-inducing ligand (TRAIL) is a member of the tumor necrosis factor (TNF) ligand family that exerts its apoptotic activity in human cells by binding to two transmembrane receptors, TRAILR1 and TRAILR2. In cells co-expressing both receptors the particular contribution of either protein to the overall cellular response is not well defined. Here we have investigated whether differences in the signaling capacities of TRAILR1 and TRAILR2 can be attributed to certain functional molecular subdomains. We generated and characterized various chimeric receptors comprising TRAIL receptor domains fused with parts from other members of the TNF death receptor family. This allowed us to compare the contribution of particular domains of the two TRAIL receptors to the overall apoptotic response and to identify elements that regulate apoptotic signaling. Our results show that the TRAIL receptor death domains are weak apoptosis inducers compared to those of CD95/Fas, because TRAILR-derived constructs containing the CD95/Fas death domain possessed strongly enhanced apoptotic capabilities. Importantly, major differences in the signaling strengths of the two TRAIL receptors were linked to their transmembrane domains in combination with the adjacent extracellular stalk regions. This was evident from receptor chimeras comprising the extracellular part of TNFR1 and the intracellular signaling part of CD95/Fas. Both receptor chimeras showed comparable ligand binding affinities and internalization kinetics. However, the respective TRAILR2-derived molecule more efficiently induced apoptosis. It also activated caspase-8 and caspase-3 more strongly and more quickly, albeit being expressed at lower levels. These results suggest that the transmembrane domains together with their adjacent stalk regions can play a major role in control of death receptor activation thereby contributing to cell type specific differences in TRAILR1 and TRAILR2 signaling.

## Introduction

Tumor necrosis factor-related apoptosis-inducing ligand (TRAIL) is a member of the tumor necrosis factor (TNF) ligand family, exerting its bioactivity on human cells *via* binding to five partners, comprising the soluble molecule osteoprotegerin (OPG) and four cell surface receptors in the human system [1]. Two of the receptors, TRAILR1 (also called DR4, APO-2 or TNFRSF10A) and TRAILR2 (DR5, TRICK2 or TNFRSF10B), are capable to activate a prominent form of programmed cell death, termed apoptosis, through their cytoplasmic death domains (DD). Two other receptors, TRAILR3 (DcR1, TRID, LIT) and TRAILR4 (DcR2, TRUNDD) may serve as decoy receptors by competitive ligand binding and/or the formation of mixed and thus non-functional ligand/receptor complexes [2]. TRAILR3 is a GPI-anchored molecule, therefore possessing no intracellular signaling domain at all, and TRAILR4 features a truncated death domain with sparsely defined signaling capabilities. OPG binds TRAIL with low affinity with unclear biological impact of this interaction [3]. To date, current research on the TRAIL system focuses on cellular responses mediated through TRAILR1 and TRAILR2. Most normal tissues are resistant to the apoptotic action of TRAIL despite cell surface receptor expression, whereas several cancer cells show remarkable sensitivity to it [4]. Therefore, TRAIL or other TRAIL receptor agonists are currently being investigated as candidates for therapeutic intervention especially for cancer treatment [5], [6].

Like most members of the TNF receptor family both apoptosis-inducing TRAIL receptors show the typical topology of many type I proteins. The extracellular C-terminal part contains three cysteine-rich domains (CRD). These CRDs form the ligand interaction site and a homophilic interaction domain at the membrane-distal region, called pre-ligand binding assembly domain (PLAD) [7]. Interestingly, and in contrast to e.g. the TNF system [8], PLAD-mediated interactions of membrane-expressed TRAILR allow homo- as well as heteromer formation, strengthening the arguments for TRAILR3 and TRAILR4 to play a role as inhibitory molecules. The respective membrane proximal CRDs are linked via so-called stalk regions to their transmembrane domains (TM). The intracellular parts contain the DD capable of binding additional DD-affine adapter proteins such as FADD (Fas associated death domain protein) [9]. Apoptotic signaling is then initiated by recruitment and autoproteolytic activation of procaspases-8 and/or -10 into the death-inducing signaling complex (DISC). However, the molecular composition of the DISC may vary depending on cell type and activation status.

Characteristic of most TNF family members, the ligand TRAIL is primarily expressed as a type 2 transmembrane protein which can be processed by proteases to release the soluble form [1]. Both, membrane-bound TRAIL (memTRAIL) and the soluble molecule (sTRAIL) form non-covalently linked homotrimers coordinated by three cysteine residues binding a central zinc ion [10]. Homotrimeric TRAIL has the capability to bind up to three receptor molecules in the grooves between its individual subunits [11]. As unligated receptors also form homo-oligomers via the PLAD, it is feasible that ligation of receptors leads to formation of larger clusters, as proposed for the TNF system recently [12], [13].

Many human cells and cell lines co-express TRAIL receptors 1 and 2 which may positively cooperate in apoptosis induction upon activation by their ligand. Whether and to what extent mixed receptor complexes contribute to signaling is unknown. In different cellular systems the overall cellular response might be dominated by either receptor [14]–[17]. Most ligand binding affinity studies demonstrate comparable values for both receptors with apparent dissociation constants in the low nanomolar range [18], [19], although much higher affinity values have been also published [20].

The aim of the present study was to investigate whether differences in the signaling capabilities of TRAILR1 and TRAILR2 can be attributed to particular domains of these molecules. For this purpose we have constructed various receptor chimeras where functional domains of TRAIL receptors had been exchanged by the corresponding domains from other death receptors. Analysis of these chimeras demonstrates that the capacity to activate apoptotic signaling is regulated not only at the level of binding partner interaction, i.e. ligand binding to the extracellular part and adapter protein binding to the intracellular part, but unexpectedly also by the respective transmembrane domains together with their adjacent stalk regions. These results suggest that processes of spatial arrangement like recruitment into particular membrane microdomains and cluster formation co-regulate sensitivity of TRAIL receptors and in particular the differential characteristics of TRAILR1 and TRAILR2.

## Results

### The death domains of TRAIL receptors are weak apoptosis inducers compared to the CD95/Fas death domain

**Figure 1:**
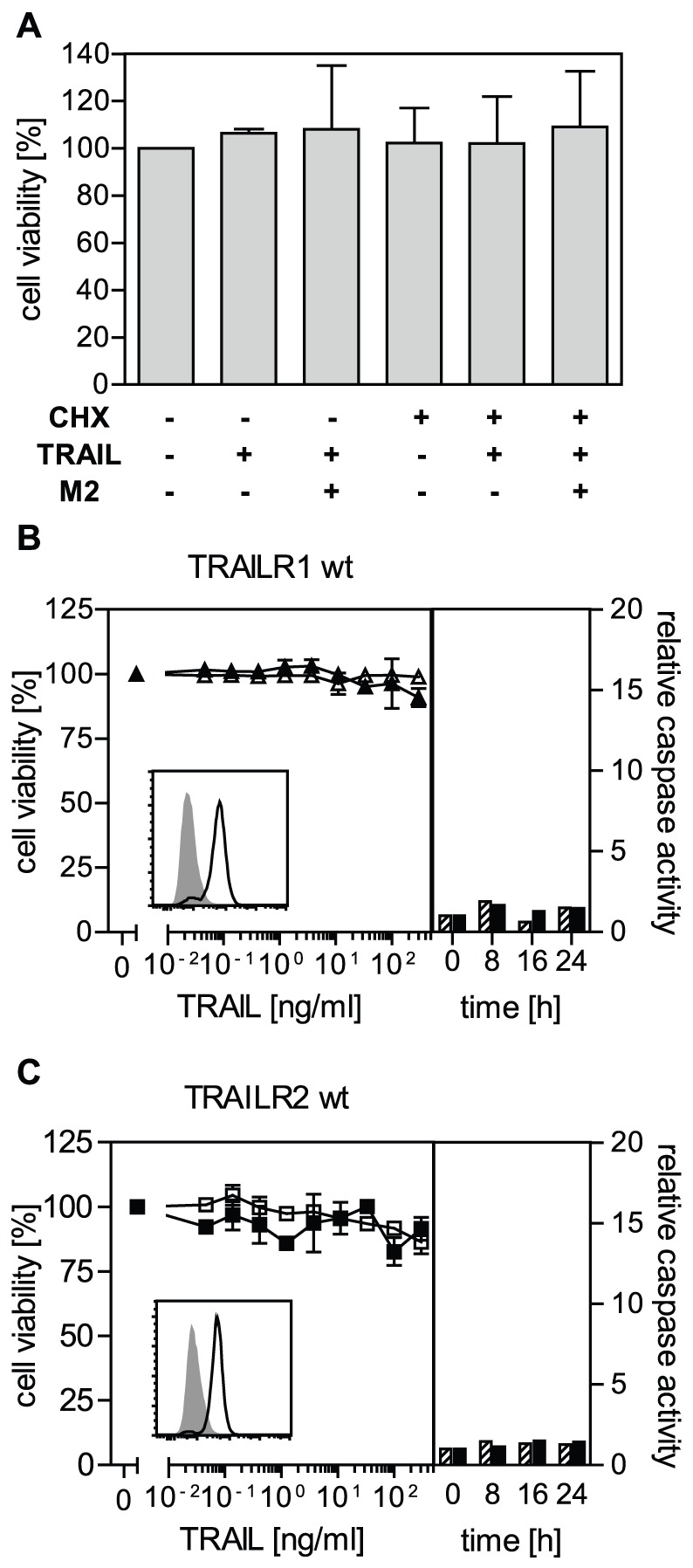
Wild type mouse-derived immortalized fibroblasts, as well as TRAILR1- and TRAILR2-positive transfectants lack sensitivity to TRAIL-induced cell death. **A.** Mouse fibroblasts are nonresponsive to the cytotoxic effects of TRAIL. Cells were treated with FLAG-TRAIL (400 ng/ml) in the absence or presence of the crosslinking anti-FLAG antibody M2 (2 µg/ml; 1 h 37°C) and/or 0.5 µg/ml cycloheximide, as indicated. Cell viability was determined by crystal violet staining the next day. One representative experiment (n = 3, shown are the mean values ± standard deviation (SD)) out of three is shown. **B and C.** Immortalized mouse fibroblasts were stably transfected with wild-type human TRAILR1 (B) or TRAILR2 (C) expression plasmids. Cell viability was determined by crystal violet staining following 16 hour incubation with cross-linked FLAG-TRAIL in the absence (open symbols) or presence (closed symbols) of cycloheximide (0.5 µg/ml). One representative experiment (n = 3, shown are the mean values ± SD) out of three is shown. Caspase-8 (dashed bars) and caspase-3 (black bars) enzymatic activity following triggering of indicated receptors with FLAG-TRAIL (previously cross-linked with anti-FLAG M2 antibody, 1 h 37°C) was quantified using specific fluorogenic substrates (Ac-IEPD-AMC and Ac-DMQD-AMC, respectively). The insets show the expression pattern of the human TRAIL receptors as analyzed by flow cytometry. One representative experiment (out of three) is shown.

In the present work we chose large T antigen-immortalized embryonic mouse fibroblasts (MF) as cellular model. These cells are unresponsive to TRAIL-mediated cytotoxic effects even in the presence of an antibody cross-linking the ligand and/or cycloheximide (CHX) which is an inhibitor of protein synthesis and known apoptosis-sensitizer (Fig. 1A). In a first approach we compared the apoptotic signaling capability of the two death domain positive human TRAIL receptors TRAILR1 and TRAILR2 which were stably expressed in MF at comparable levels (Fig. 1B and 1C, insets). No cytotoxic response was observed after stimulation with TRAIL under various conditions, including the presence of CHX and/or the use of a ligand-specific antibody to increase the potency by secondary cross-linking. In addition, no significant activation of caspase-8 or -3 could be observed within a time frame of 24 hours (Fig. 1B and 1C). As we have previously shown that Fas and TNFR-Fas chimeras are rapid and strong apoptosis inducers in MF (data not shown, [21]), these results demonstrate that significant differences must exist between signal initiation of TRAIL receptors and Fas.

**Figure 2:**
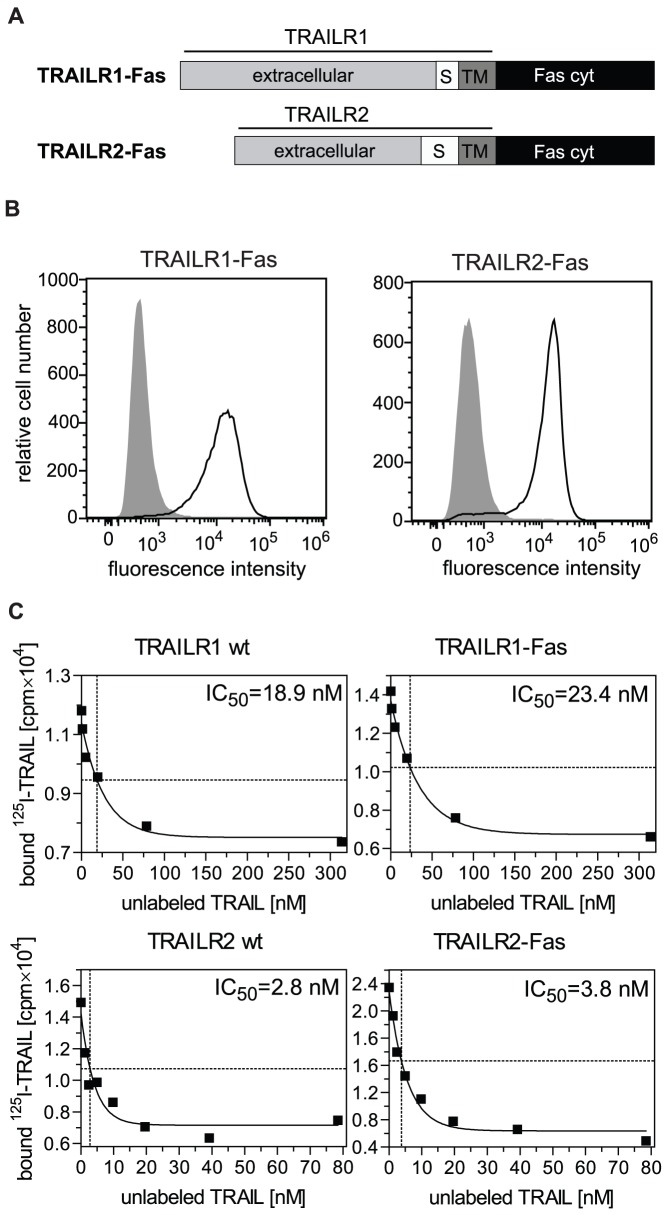
TRAILR1-Fas and TRAILR2-Fas chimeric receptors show ligand binding affinities comparable to the respective wild-type TRAIL receptors. **A.** Schematic representation of TRAILR1-Fas and TRAILR2-Fas chimeric proteins. The cytoplasmic domain of Fas (Fas cyt), amino acids 191–335, was fused to the C-terminus of the potential transmembrane region of TRAILR1 (amino acid 262) or TRAILR2 (amino acid 231). S = stalk region, TM = transmembrane domain. **B.** Immortalized mouse fibroblasts were stably transfected with TRAILR1-Fas and TRAILR2-Fas expression plasmids. Cell surface expression was analyzed by flow cytometry using TRAILR1- and TRAILR2-specific antibodies. Isotype controls are shown in grey. **C.** Representative curves from ligand binding competition experiments on wild-type (wt) TRAILR positive cells and TRAILR-Fas chimera expressing cells using ^125^I-labeled sTRAIL. IC_50_-values determined from ligand binding competition studies indicate differential affinities of the ligand towards TRAILR1(-Fas) *versus* TRAILR2(-Fas) chimeric receptors.

We next constructed and characterized TRAILR-Fas chimeras by replacing the intracellular parts of TRAIL receptors 1 and 2 containing the DD with that of Fas (Fig. 2A). As shown by flow cytometry these receptor chimeras were expressed in MF at comparable and high levels (Fig. 2B). We first studied ligand binding affinities of both TRAILR-Fas chimeras in comparison to the respective wildtype TRAIL receptors by equilibrium binding competition studies using radioiodinated TRAIL. Comparable affinity values were obtained for the respective wild-type and chimeric receptor pairs by similar displacement rates with non-labeled TRAIL. Whereas the IC_50_–value for TRAILR1 (chimera) was around 17 nM, TRAILR2 (chimera) showed a significantly higher ligand binding affinity with approximately four-fold lower IC_50_-values (representative results are shown in Fig. 2C) (TRAILR1: IC_50_ = 17.7±4.3 nM, TRAILR1-Fas: IC_50_ = 16.8±6.7 nM; TRAILR2: IC_50_ = 3.5±2.8 nM, TRAILR2-Fas: IC_50_ = 5.5±1.8 nM; mean values ± SD, n = 3). These data indicate that the exchange of the intracellular signaling parts had not affected ligand/receptor interaction. The obtained IC_50_-values in the lower nanomolar range are in accordance with dissociation constant values from literature data [18], [19] and overlap with the concentration range of 100–800 ng/ml of TRAIL used in our experimental studies.

**Figure 3:**
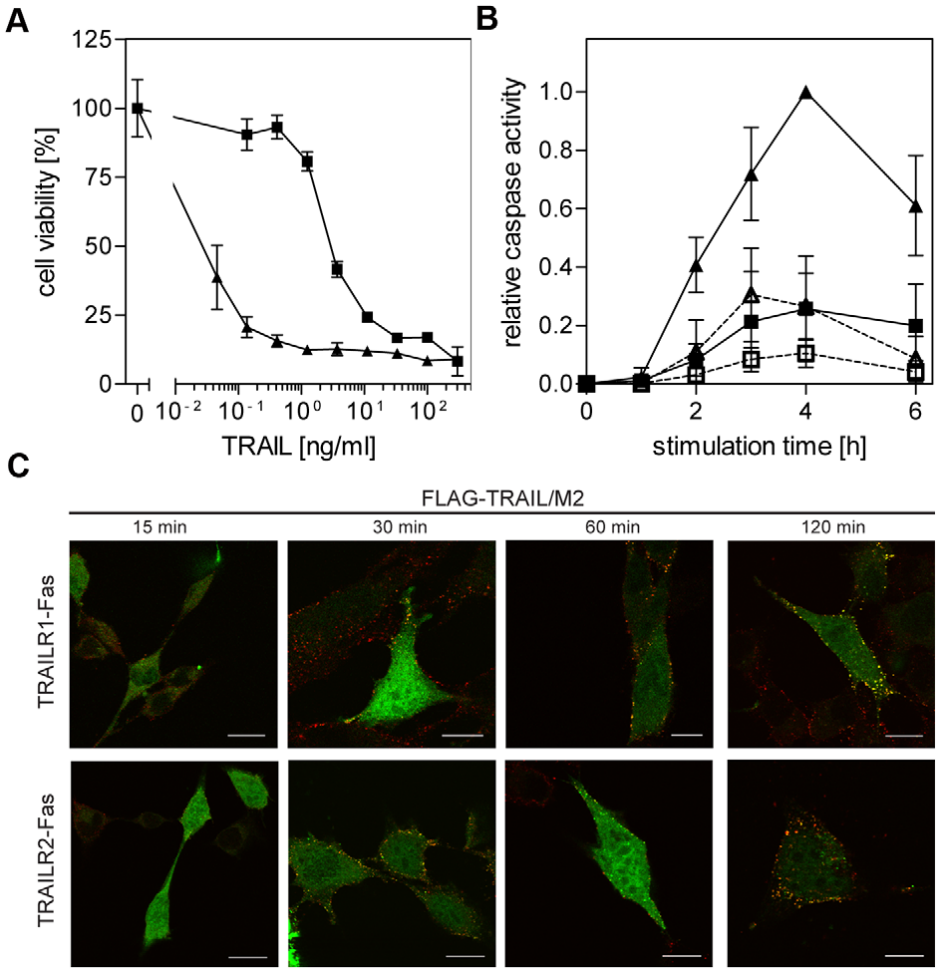
Differential TRAIL responsiveness, but comparable ligand/receptor cluster formation of TRAILR1-Fas and TRAILR2-Fas receptor chimeras. **A.** Cytotoxic effects of TRAIL in TRAILR1-Fas and TRAILR2-Fas expressing mouse fibroblasts. MF-TRAILR1-Fas (closed squares) or MF-TRAILR2-Fas cells (closed triangles) were treated with serial dilutions of FLAG-TRAIL (previously cross-linked with anti-FLAG M2 antibody, 1 h 37°C) in the presence of cycloheximide (0.5 µg/ml). Cell viability was determined by crystal violet staining the next day. All experimental groups shown were performed in parallel, one representative experiment out of three is shown (performed in triplicates, shown are mean values ± SD). **B.** Caspase-8 (open symbols, dashed lines) and caspase-3 (closed symbols, solid lines) enzymatic activities following stimulation of MF-TRAILR1-Fas or MF-TRAILR2-Fas (squares and triangles, respectively) with 100 ng/ml FLAG-TRAIL (previously cross-linked with 2 µg/ml anti-FLAG M2 antibody, 1 h 37°C) were determined using specific fluorogenic substrates (Ac-IEPD-AMC and Ac-DMQD-AMC, respectively). Data points are mean values ± SD calculated from three independent experiments and normalized using the highest activity value (caspase-3 activity in MF-TRAILR2-Fas after 4 hours of stimulation). **C.** MF-TRAILR1-Fas and MF-TRAILR2-Fas cells were transiently transfected with a FADD-eGFP construct in the presence of 20 µM z-VAD-fmk. The following day cells were treated with FLAG-TRAIL (300 ng/ml, previously cross-linked with 2 µg/ml mouse anti-FLAG M2 antibody, 1 h 37°C) for the indicated time periods. After fixation cells were stained with an Alexa Fluor 546-labeled anti-mouse IgG antibody and examined by confocal laser-scanning microscopy. An optical section through the center of one representative cell for each time point is shown.

In contrast to the respective wild-type TRAIL receptors, both TRAILR-Fas chimeras were capable to induce strong cytotoxic responses. In MF positive for TRAILR1-Fas (MF-TRAILR1-Fas cells) the presence of CHX was necessary to allow a strong cytotoxic response to soluble TRAIL, whereas MF-TRAILR2-Fas cells were highly responsive even in the absence of secondary crosslinkers and metabolic inhibitors (Fig. S1). In the presence of CHX and the antibody M2 used to cross-link the FLAG-tagged ligand both cell lines showed strong cytotoxic responses at nanomolar TRAIL concentrations (Fig. 3A), which could be fully inhibited using the broad-range caspase inhibitor z-VAD-fmk (data not shown). Importantly, dose response curves revealed different sensitivities, with the TRAILR2-derived chimera showing an approximately 100-fold higher sensitivity based on the half-maximum effector concentration. At a concentration of 1 ng/ml of TRAIL a near to maximum cytotoxic effect was observed in TRAILR2-Fas positive cells, whereas TRAILR1-Fas positive MF showed no response at all (Fig. 3A). Similar differences were obtained also for ligand stimulation in the absence of a secondary crosslinker (Fig. S1). Stronger cytotoxic responses of TRAILR2-derived chimeras were paralleled by a two- to three-fold stronger activation of caspases-8 and -3 (Fig. 3B). Overall activation kinetics of both caspases was comparable reaching half of their maximum activity after about two hours of ligand stimulation and their maximum values after four hours. The higher ligand binding affinity of the TRAILR2-derived chimera as shown in Figure 2C might contribute to its higher apoptotic capacity paralleled by more efficient caspase activation (Fig. 3A and B). It appears unlikely, however, that approximately a fourfold difference in ligand binding affinity is responsible for a sensitivity shift of almost two orders of magnitude. Accordingly, substituting the death domain of TRAIL death receptors with the corresponding domain of CD95/Fas yielded chimeric death receptors with considerably enhanced apoptotic signaling capabilities and profound differences in their signaling strengths. As the slight difference in their ligand binding affinities cannot explain the observed divergent signaling strengths, the existence of additional mechanisms regulating TRAIL receptor signal initiation is likely.

### FADD is recruited to the death domains of TRAILR1-Fas and TRAILR2-Fas chimeras

We also investigated receptor cluster formation and FADD recruitment to the Fas-derived death domains of the receptor chimeras by confocal microscopy visualizing colocalized FADD-eGFP with Alexa Fluor 546-stained ligand. First clusters became visible after about 30 minutes of ligand stimulation. We observed similar kinetics in further cluster growth resulting in comparable and strong cluster formation after about two hours (Fig. 3C). Thus, cluster formation clearly preceded the observed strong activation of caspase-8 (Fig. 3B). As in several experiments, however, observers had the impression that TRAILR2-derived receptors showed a somewhat faster cluster formation kinetics than their counterparts, we quantitatively analyzed colocalization of ligand with FADD-eGFP in MF-TRAILR1-Fas and MF-TRAILR2-Fas cell lines, respectively, at different time points after stimulation (Fig. S2). The results obtained are consistent with a faster cluster formation in TRAILR2-Fas expressing cells, leading to comparable clustering after longer stimulation. Yet this analysis also revealed a high cell to cell variance of FADD and TRAIL colocalization, thus the differences in dynamics of cluster formation are statistically significant only for the 15 min time-point of stimulation.

### Transmembrane domain and adjacent sequences contribute to the difference in TRAILR1 and TRALR2 signaling competence

**Figure 4:**
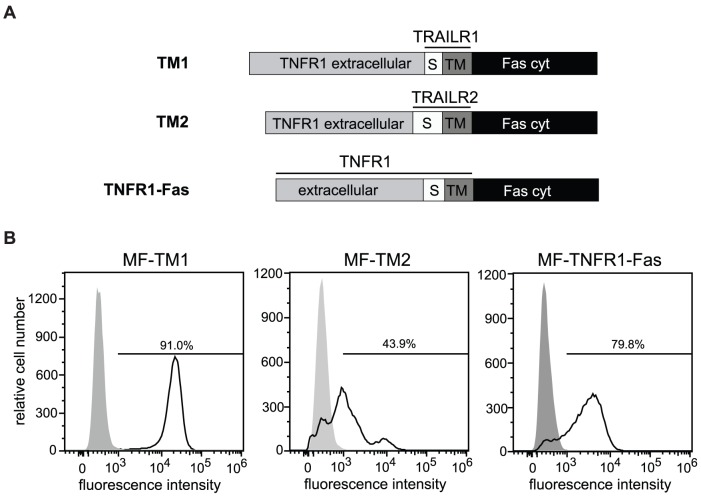
Receptor chimeras with identical ligand binding sites and intracellular signaling domains. **A.** Schematic representation of TNFR1-TRAILR1-Fas (TM1), TNFR1-TRAILR2-Fas (TM2) and TNFR1-Fas chimeric proteins. TNFR1-Fas receptor was established through the fusion of the cytoplasmic domain of Fas, amino acids 191–335, to the C-terminus of the potential transmembrane region of TNFR1 (amino acid 236). In TM1 and TM2 receptor chimeras the stalk (S) and transmembrane (TM) regions of TNFR1 (aa 197–234) were then substituted with the corresponding regions of TRAILR1 (amino acids 230–262) or TRAILR2 (amino acids 179–231). **B.** Immortalized mouse fibroblasts were stably transfected with TM1, TM2 and TNFR1-Fas expression plasmids. Expression of the chimeras was analyzed by flow cytometry using TNFR1-specific antibodies. Percentage of positive cells is indicated.

The apoptotic activity of TRAIL receptors has been associated with their localization in cholesterol-rich microdomains, formerly often called lipid rafts [22], [23]. Targeting of signaling molecules into microdomains is likely to be regulated, among others, by the transmembrane part (TM) of the receptor itself and adjacent sequences. For TRAILR1 (cysteine residue(s) 161–163; [22]) and the Fas molecule [24], but not TRAILR2, palmitoylation has been demonstrated as a driving factor for partitioning within cellular membranes. To investigate if the TMs of TRAIL receptors influence their differential strengths in apoptosis signaling, new chimeric molecules were generated: The extracellular ligand binding domains were derived from TNFR1 and their cytoplasmic signaling domains were Fas-derived. However, transmembrane domains and the extracellular stalk regions were derived from TRAIL receptors 1 or 2 (see schematic drawing Fig. 4A). The stalk regions are different in length comprising about 10 amino acids (aa) for TRAILR1 and about 34 aa for TRAILR2.

**Figure 5:**
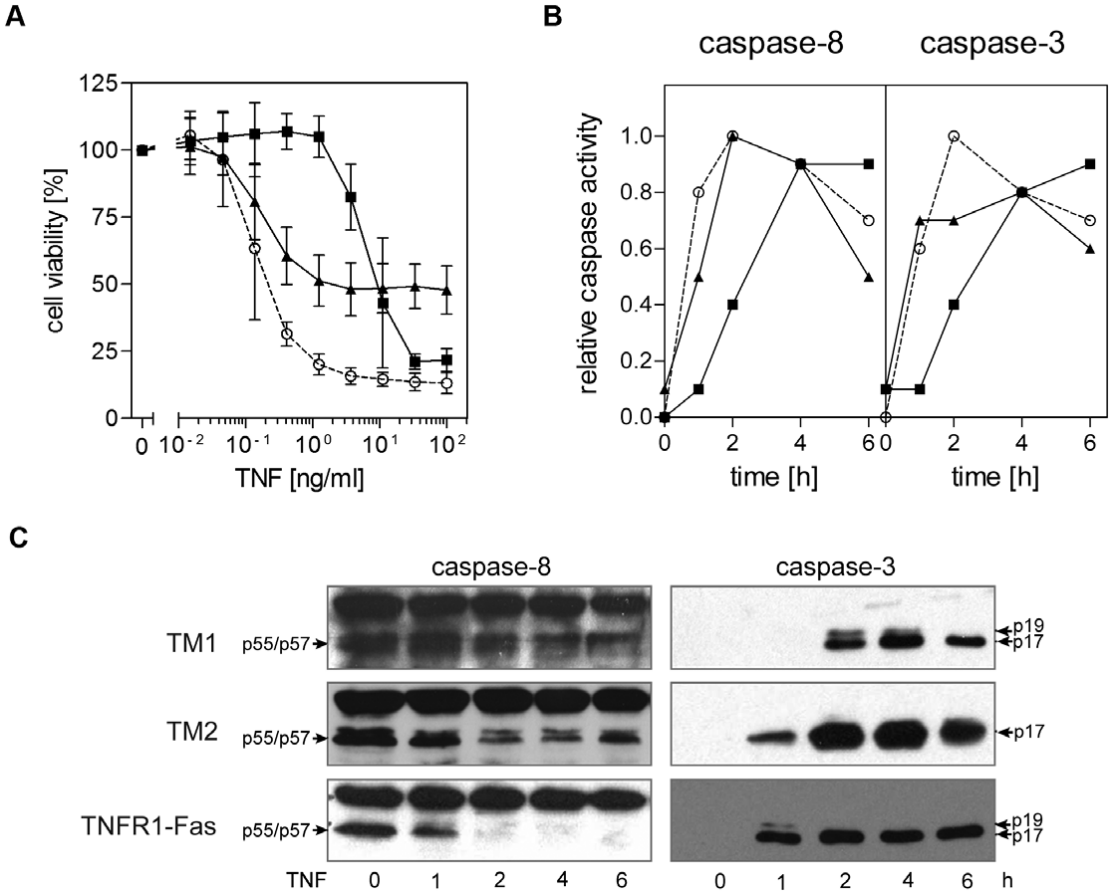
The transmembrane and stalk regions of TRAILR1 and TRAILR2 regulate apoptotic signaling. **A.** Cytotoxic effects of TNF in TM1, TM2 and TNFR1-Fas expressing mouse fibroblasts. MF-TM1 (closed squares), MF-TM2 (closed triangles) or MF-TNFR1-Fas (open circles) cells were treated with serial dilutions of TNF and cell viability was determined by crystal violet staining after 6 hours of stimulation. All experimental groups shown were performed in parallel: one representative experiment out of three is shown. **B.** Caspase-8 (left panel) and caspase-3 (right panel) enzymatic activities following triggering of TM1, TM2 and TNFR1-Fas (closed squares, closed triangles and open circles, respectively) positive cells with TNF were determined using specific fluorogenic substrates (Ac-IEPD-AMC and Ac-DMQD-AMC, respectively). Data from a representative experiment out of three are shown. The graphs depict normalized caspase activities (maximum relative activity = 1), as absolute activity values are likely to be strongly influenced by the differential expression levels of the chimeric receptors. **C.** Western blot analyses performed using procaspase-8- and cleaved caspase-3-specific antibodies. Cells stably expressing chimeric receptors were treated with TNF (100 ng/ml) for the indicated times, followed by cell lysis.

Despite the novel chimeric receptors were identical in both their ligand binding and signaling domains significant differences were observed. MF positive for the construct containing the TM and stalk region of TRAILR2 (MF-TM2) showed heterogeneity at the level of expression with an overall lower cell surface expression as determined by flow cytometry (Fig. 4B). In spite of these reduced levels at the cell surface MF-TM2 cells showed a significantly higher TRAIL sensitivity. Near to maximum cytotoxic effects were observed at TNF concentrations above 1 ng/ml, whereas MF-TM1 cells, expressing the construct containing the TM and stalk region of TRAILR1, were unresponsive at these ligand concentrations (Fig. 5A). Maximum cell death in MF-TM2 cells did not affect more than about 50% of all cells (Fig. 5A), but this is readily explained by the heterogeneous and on average lower expression of the TM2 receptor chimera. In fact, MF-TM2 cells showed a sensitivity comparable to MF-TNFR1-Fas cells, containing the TM and stalk region of TNFR1 [21], which had been included for comparison. Notably, strong cytotoxic responses could be initiated by the soluble ligand TNF in the absence of a secondary crosslinker as well as CHX. When we investigated kinetics of caspase activation we observed in MF-TM2 cells a faster onset of caspase enzymatic activity (Fig. 5B) as well as enzyme cleavage (Fig. 5C) for both the initiator caspase-8 and the effector caspase-3. MF-TRAILR1-Fas (Fig. 3B) and MF-TM1 cells (Fig. 5B) revealed comparable activation kinetics for both caspase-8 and caspase-3, with 50% of their activity after approximately two hours of ligand stimulation. MF-TM2 cells, on the other hand, showed faster caspase activation kinetics (50% of caspase activity after approximately 1 hour) in comparison to both MF-TM1 and MF-TRAILR2-Fas cells (compare Fig. 5B and Fig. 3B). Processing of the effector caspase-3 could be detected in MF-TM2 cells as early as after one hour of ligand stimulation. In contrast, in identically treated MF-TM1 cells cleavage of caspase-3 was only observed after one additional hour of TNF stimulation (Fig. 5C). These data indicate that the TRAIL receptor-derived TM regions and/or adjacent sequences such as the stalk regions impact on signaling kinetics and/or strengths.

### Transmembrane domains and adjacent sequences only marginally impact ligand-binding affinities of TM1 and TM2 chimeric receptors

**Figure 6:**
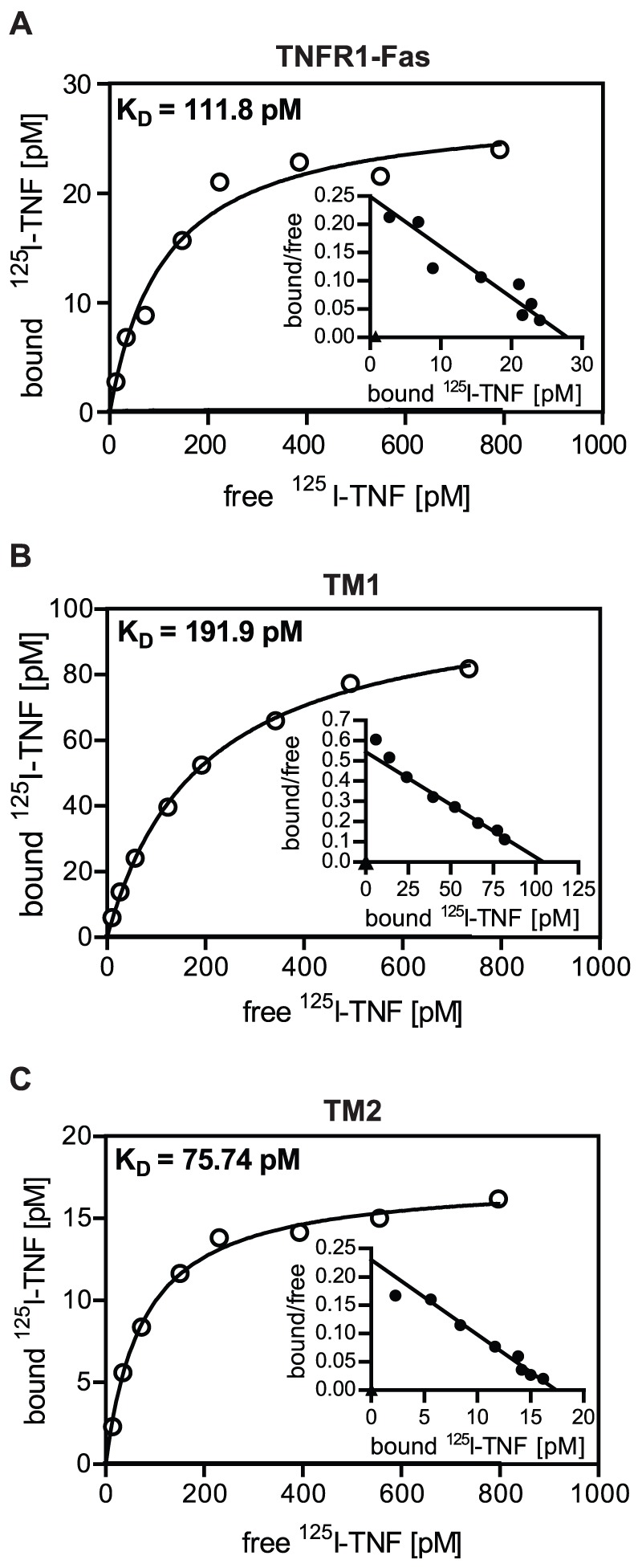
Ligand binding affinities of receptor chimeras differing only in their transmembrane and stalk regions. **A-C.** Mouse fibroblasts expressing the indicated chimeric receptors (2×10^5^ cells per sample) were incubated with radioiodinated TNF at different concentrations for 2 hours on ice. Cells were separated from unbound label by centrifugation through phthalate oil and cell bound protein was quantified. Free radioactive TNF was plotted against bound label, a one site binding hyperbola was fitted through the data points and K_D_-values were determined by using the Graphpad Prism software. Data points represent mean values out of duplicates. The insets show Scatchard plots of the corresponding data.

TNF and TRAIL both form homotrimeric molecules capable of binding up to three receptors [11], [13]. In addition all four TRAIL membrane receptors are able to homomultimerize in the absence of ligand *via* the aforementioned PLAD [7], [25]. It is therefore feasible that in binding studies, performed under conditions of reduced membrane fluidity, ligand/receptor interactions occur with different stoichiometries resulting in distinct effective affinities caused by avidity effects. We therefore performed equilibrium binding studies with radioiodinated TNF revealing comparable ligand binding affinities for the two receptor chimeras (225±17.2 nM for TM1 and 71±4.3 nM for TM2; mean K_D_-values ± SD of 3 experiments each) as well as for TNFR1-Fas (112±10.8 nM; n = 3). This finding argues against major differential avidity effects. Consistently, however, MF-TM2 cells showed an about threefold lower value for the apparent dissociation constant compared to MF-TM1 cells (see also the example shown in Fig. 6). Binding data had been fitted to a one site binding hyperbola and the linearity of the respective Scatchard analyses (Fig. 6A–C, insets) indicates good agreement with this assumption. No indications are visible in these diagrams for the existence of e.g. two distinct affinities. It is therefore likely that minor conformational changes within the chimeric molecules cause the slight affinity differences observed. In any case the observed differences in ligand binding affinities appear too small to explain the differential apoptotic capabilities observed. Additional constraint(s) in TM1 and TM2 molecules must exist, most likely located within the TM regions and/or the adjacent stalk regions, which efficiently regulate ligand sensitivity.

### TM1 and TM2 show comparable dynamics of FADD recruitment and internalization after ligand binding

**Figure 7:**
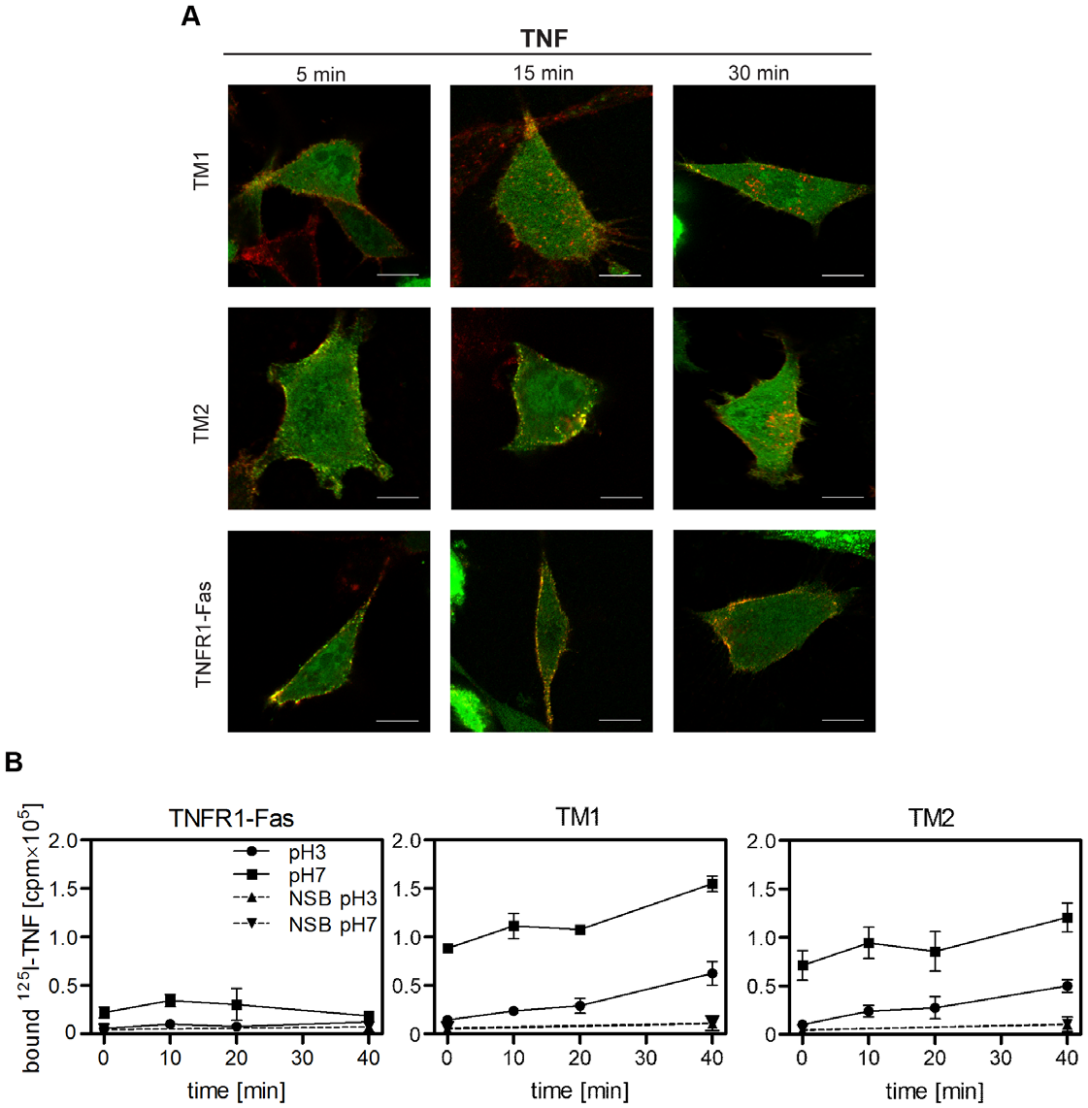
Receptor chimeras with the transmembrane and stalk regions from TRAILR1 and TRAILR2 show comparable ligand-induced receptor aggregation and internalization. **A.** MF-TM1, MF-TM2 and MF-TNFR1-Fas cells were transiently transfected with a construct expressing human FADD-eGFP in the presence of 20 µM z-VAD-fmk. The following day cells were treated with Alexa Fluor 546-labeled TNF (100 ng/ml) for the indicated time periods, fixed and examined by confocal laser-scanning microscopy. Shown are optical sections through the center of representative cells. **B.** Adherent cells were incubated with ^125^I-TNF (30 ng/ml) for 1 h on ice, followed by incubation at 37°C and 5% CO_2_ for the indicated times. Subsequently cells were washed with PBS, followed by washing with acidic buffer (pH = 3.0) to disrupt ligand/receptor interactions on the cell surface, or again with PBS (pH = 7.0). For non-specific binding (NSB) a 200-fold excess of unlabeled TNF was added during the first incubation step. Radioactivity of the cell lysates was then quantified in a γ-counter. Data points represent mean values ± SD from three independent experiments each performed in duplicates.

It is currently accepted that TNFR1 forms two distinct, subsequently assembled signaling complexes of which the secondary, internalized complex is capable to activate caspase-8 [26], [27]. In contrast, ligand activated TRAIL death receptors are believed to signal apoptosis induction both via a membrane-associated primarily formed DISC, and from intracellular ubiquitin-rich foci formed after polyubiquitylation of caspase-8 [9]. We therefore compared receptor cluster formation and internalization of the receptor chimeras after TNF stimulation. Mouse fibroblasts positive for TM1, TM2 or TNFR1-Fas were transiently transfected with a construct expressing human FADD-eGFP. The next day cells were treated with Alexa Fluor 546-labeled TNF (100 ng/ml) for the indicated time periods at 37°C, fixed and examined by confocal laser-scanning microscopy. The examples shown in Fig. 7A are optical sections through the center of the cells and demonstrate comparable cluster formation of the two chimeras comprising the TRAILR1- and TRAILR2-derived transmembrane parts. Additionally, they indicate significant internalization of the chimeras in contrast to TNFR1-Fas chimeras, where internalized clusters were hardly visible.

To study ligand/receptor complex internalization more directly, cells were allowed to bind radioiodinated TNF at 0°C, representing conditions where receptor internalization is largely inhibited, and were then incubated at 37°C to allow internalization. At different times cells were washed at pH 7.0 to reveal total bound radioactivity or at pH 3.0. At this acidic pH value cell surface bound ligand is released from the receptors, whereas internalized material remains cell-associated. TNFR1-Fas positive cells were included as controls as they internalize ligated receptors only very slowly (data not shown). Unspecific binding was quantified in the presence of a 200-fold excess of unlabeled TNF. The results clearly demonstrate that ^125^I-TNF had specifically bound to the cells after the incubation period (t = 0), and could effectively be removed by washing the cells at pH 3.0 (Fig. 7B). With increasing incubation times at 37°C an increasing part of the radiolabeled TNF remained cell-associated, which was interpreted as internalized receptor chimeras. TNFR1-Fas positive control cells showed a reduced ligand binding capacity and significant receptor internalization was not observed. The decrease in total binding of these control cells after 20 minutes is likely to be caused by ongoing apoptosis, known to occur in TNFR1-Fas cells very rapidly [21]. Together, these results strongly suggest that the two receptor chimeras become internalized after ligand binding at a similar rate.

## Discussion

In this study we have obtained three major results regarding the two death receptors of the human TRAIL system. First of all, the death domains of TRAILR1 and TRAILR2 transduce the apoptotic signal less efficiently than the corresponding domain of the death receptor Fas. This is also evident from the resistance to TRAIL stimulation of most normal tissue cells but also many tumor cells [4], [28]. In accordance with these data, large T antigen immortalized mouse fibroblasts positive for wild-type human TRAIL receptors showed no significant cytotoxic responses upon ligand stimulation, even in the presence of secondary ligand crosslinkers and/or metabolic inhibitors (Fig. 1). Crosslinked ligand, however, induced a significant activation of the transcription factor NF-κB, as well as recruitment of huFADD-eGFP fusion proteins into ligand/receptor complexes (data not shown), indicating that both human receptors were functional also in a mouse background. Furthermore, immortalized mouse fibroblasts expressing human TNFR1-Fas receptor chimeras had been previously shown to effectively activate the apoptotic program [21]. Replacing the intracellular signaling parts of TRAILR1 and TRAILR2 by the corresponding domain of Fas improved the signaling capability strongly (compare Fig. S1 and Fig. 3). The resulting TRAILR-Fas chimeras were capable of inducing strong apoptotic responses and revealed affinity values identical to those of the respective wild-type receptors in ligand binding competition studies (Fig. 2C). These results strongly suggest major differences in the death domain signaling capabilities between the two TRAIL receptors and Fas, although all three molecules are believed to induce apoptosis via FADD recruitment [9], [29].

The second important finding of this work is that the two TRAILR-Fas chimeras showed significant differences in their signaling strengths, which could neither be attributed to their intracellular signaling parts, as these were identical, nor to diverging ligand binding affinities, as ligand binding competition studies revealed only a difference of a factor of approximately four. Significant differences in their efficiency in apoptotic signaling have been reported to occur between wild-type TRAILR1 and TRAILR2. This aspect has gained attention in the scientific community because many tissues co-express the two death domain-containing TRAIL receptors and TRAIL is considered to possess a high therapeutic potential in particular for cancer therapy [5], [6]. In some cell lines TRAILR1 dominated the apoptotic response [14], [16], [17], yet in other experimental systems the apoptotic response was strongly dependent on TRAILR2, despite high levels of cell surface expression of TRAILR1 [15]. However, in most publications it was not taken into account that TRAILR2, but not TRAILR1, is strongly dependent on the membrane bound ligand form for full activation [30], [31]. The anti-FLAG M2-closslinked FLAG-tagged TRAIL used in this study efficiently mimics the particular bioactivity of membrane bound TRAIL, resulting in full activation of both receptors. Together, the TRAILR2-derived chimera displays a TRAIL responsiveness which is higher by approximately two logs as compared to the respective TRAILR1-derived chimera, although both molecules possess the identical Fas-derived signaling part and show ligand binding affinities with differences of only about a factor of four.

To further minimize non-identical domains we replaced the extracellular ligand binding domains of the receptor chimeras with that of TNFR1. This resulted in molecules identical in both their extracellular ligand binding parts and their intracellular signaling domains, but different in their TM domains and the adjacent stalk regions, i.e. the sequences connecting the TM with the cysteine rich domains. Remarkably, comparison of these two molecules still revealed profound differences, which is the third major result of this study. Although showing reduced and inhomogeneous plasma membrane expression, receptor chimera TM2, comprising TM and stalk of TRAILR2, was still a significantly faster and stronger activator of caspases and inducer of apoptosis than TM1 (Fig. 5). Nevertheless, we observed comparable signaling cluster formation (Fig. 7A) and kinetics of receptor cluster internalization in both cell lines (Fig. 7B). Again, one obvious mechanism resulting in higher TM2 responsiveness could result from higher affinity in ligand/receptor interaction. However, as derived from equilibrium binding studies with radio-iodinated TNF, binding affinities of the receptor chimeras TM1 and TM2 were very similar showing only an approximately threefold difference in their apparent K_D_-values (Fig. 6). Moreover, the TNF concentration of 100 ng/ml which was used in the experiments is one order higher than the apparent K_D_-values of these molecules, which ensures that ligand binding conditions are near to saturation. Under these conditions the observed threefold difference in affinities between TM1 and TM2 receptor chimeras should have no significant impact on the respective signaling strengths. Accordingly, we conclude that additional molecular mechanisms caused by distinct TM together with the adjacent stalk regions will control kinetics of caspase activation (Fig. 5B and C) and strength of the elicited apoptotic signal (Fig. 5A).

Cluster formation of ligated, but also multimerisation of unligated receptors is likely to be co-regulated by the composition of the cell membrane being different in distinct membrane microdomains. The TM regions of TRAILR1 and TRAILR2 have comparable predicted lengths of 23 and 21 amino acids, but the TM of TRAILR1 carries a S-palmitoylation site within the cysteine triplet Cys261-Cys263, also present in the respective receptor chimeras. S-palmitoylation favors localization of TRAILR1 in cholesterol rich microdomains (“lipid rafts”) and was shown to enhance its apoptotic activity [22], which is likely to occur *via* ligand-independent enrichment/preclustering of these molecules in membrane microdomains, allowing better responsiveness particularly to the soluble ligand. Another difference between the two TM regions, which may contribute to the formation of stable TRAILR2 homodimers, is the existence of a GXXXG motif (aa 212–216 = GIIIG), which is absent in TRAILR1. GXXXG motifs are known to serve as dimerization motifs of helical TMs and have been extensively studied in glycophorin A and the Erb B receptor [32]. However, whether this motif plays a role in TRAILR2, and therefore also in TM2 chimeras, awaits further investigation. In summary, the present study sheds light on the molecular mechanisms behind the observed differential apoptotic capabilities of the two TRAIL receptors TRAILR1 and TRAILR2. Our results demonstrate that the respective transmembrane domains together with their stalk regions have differential regulatory properties on ligand-induced apoptotic signaling. These results suggest that the local composition of the plasma membrane could have a regulatory function in TRAIL death receptor signaling and would finally allow the cell to control TRAIL sensitivity even in a spatial manner on the cell surface.

## Materials and Methods

### Plasmids and polymerase chain reactions

Expression plasmids encoding the fusion proteins TRAILR1-Fas and TRAILR2-Fas were produced by PCR cloning. The extracellular and transmembrane domains of human TRAILR1 or TRAILR2, respectively, were amplified by PCR introducing KpnI restriction sites at the 5′ and 3′ ends. This fragment was then inserted into the previously described pBSTNFR1-Fas plasmid [21], substituting the extracellular and transmembrane domain of TNFR1 by the respective domains of TRAILR or TRAILR2 and yielding the plamids pBSTRAILR1-Fas and pBSTRAILR2-Fas.

The constructs encoding the chimeric TNFR1-TRAILR1-Fas (TM1) and TNFR1-TRAILR2-Fas (TM2) receptors were generated by site-directed mutagenesis and molecular cloning from the cloning vectors pBSTRAILR1-Fas, pBSTRAILR2-Fas and pBSTNFR1-Fas. An additional BamHI endonuclease restriction site was integrated into the plasmid pBSTRAILR1-Fas between the membrane proximal cysteine-rich domain (CRD3) and the stalk region. Subsequently, the fragment encoding intracellular, transmembrane and stalk region of TRAILR1-Fas or TRAILR2-Fas, respectively, were cloned in frame into pBSTNFR1-Fas by (partial) digestion with BamHI. All constructs generated by PCR were verified by sequencing and subsequently subcloned into the expression vector pEFpgkpuroA [33] using the restriction endonucleases BamHI and EcoRV (New England Biolabs Inc.), yielding the expression constructs pEFpuroTRAILR1-Fas, pEFpuroTRAILR2-Fas, pEFpuroTNFR1-TRAILR1-Fas and pEFpuroTNFR1-TRAILR2-Fas. The plasmid pFADD-eGFP was a kind gift from Michael Lenardo (National Institutes of Health, Bethesda, MD).

### Reagents

Recombinant human TNF (2×10^7^ U/mg) was kindly provided by Knoll AG (Ludwigshafen, Germany). Flag-tagged recombinant human TRAIL (sTRAIL) was purchased from Axxora (Lörrach, Germany). Na-^125^I was purchased from Hartmann Analytic GmbH (Braunschweig, Germany). Cycloheximide was from Sigma Aldrich Chemie GmbH (Taufkirchen, Germany). All chemicals were of analytical grade.

### Western blotting

Samples were resolved by Tris/glycine SDS-PAGE and transferred onto nitrocellulose membrane (Whatman, Schleicher & Schüll, Dassel, Germany), which were then blocked with Roche blocking reagent or 7.5% non-fat milk powder in 0.01% (v/v) Tween-20/PBS for 1 hour at room temperature. Procaspase-8 (p55/p57) was detected using α-murine caspase-8 (CellSignaling Technology Inc, Danvers, USA) antibody, for detection of cleaved caspase-3 α-cleaved caspase-3 (CellSignaling) was used together with α-rabbit IgG (H+L), HRP-conjugated secondary antibody (Dianova, Hamburg, Germany).

### Cell culture and cell death assays

Murine fibroblasts (MF) generated from TNFR1/TNFR2 double knockout mice and stably transfected with human TNFR1-Fas (MF-TNFR1-Fas) have been described elsewhere [21]. Immortalized mouse fibroblasts were grown in RPMI 1640 medium containing 2 mM L-glutamine (Invitrogen) supplemented with 5% (v/v) heat-inactivated fetal calf serum (PAN Biotech, Aidenbach, Germany). For cell death assays mouse fibroblasts (1×10^5^ cells/well) were grown in 96-well plates overnight. The next day cells were stimulated with serial dilutions of recombinant human sTNF or sTRAIL (the latter previously crosslinked by incubation with 2 µg/ml anti-FLAG M2 antibody for 1 hour at 37°C). Following overnight (approx. 18 hours) incubation, cells were stained with crystal violet (20% methanol, 0.5% crystal violet) and optical density at 550 nm was determined with an ELISA plate reader as described [21].

### Binding kinetics

Equilibrium binding studies were performed as described [21]. Briefly, TNF and TRAIL were labeled with ^125^I with the use of iodobeads (Pierce, Thermo Fisher Scientific, Bonn, Germany). Murine fibroblasts expressing the respective receptor (2×10^5^ cells) were resuspended in PFA (PBS, 2% FCS, 0.002% sodium azide). Cells were incubated with increasing concentrations of ^125^I-TNF (0.25–12.5 ng/ml) for 2 h on ice. Non-specific binding was determined in the presence of a 200-fold excess of unlabeled protein. Cell-bound ^125^I-TNF was determined after centrifugation of the cells through a phthalate oil mixture. For calculations of the binding affinities a molecular mass of 51 kDa for TNF was used.

Binding competition studies using ^125^I-labeled TRAIL were performed as follows. Murine fibroblasts expressing the respective (chimeric) TRAIL receptor (4×10^5^ cells) were resuspended in PFA and incubated with 83 ng/ml ^125^I-TRAIL in the presence or absence of unlabeled recombinant human TRAIL (83–5300 ng/ml) for 4 hours on ice. Cell bound ^125^I-TRAIL was then quantified as described above. For calculation of the IC_50_-value a molecular mass of 68 kDa for TRAIL was used.

### Caspase activity assay

3×10^5^ cells were seeded in 6-well plates and allowed to grow overnight. Stimulation was performed by addition of 100 ng/ml recombinant human sTRAIL (crosslinked by pre-incubation with 2 µg/ml FLAG-specific M2 antibody) or 10 ng/ml TNF for the indicated time periods. Subsequently, cells were harvested, pelleted by centrifugation and the cell pellet was washed with ice-cold PBS. Cell lysis was performed by incubation with caspase lysis buffer (100 mM Tris-HCl pH 7.4, 200 mM NaCl, 1% NP-40, 1 mM DTT, complete® protease inhibitor (Roche Diagnostics GmbH, Mannheim, Germany)). Protein concentration was determined by Bradford assay (Bio-Rad Laboratories GmbH, Munich, Germany). For caspase-8 activity assays 20 µg, for caspase-3 assays 2 µg of total protein were used. Cell lysates were then incubated with caspase activity buffer (10 mM HEPES (pH 7.4), 220 mM mannitol, 68 mM sucrose, 2 mM NaCl, 2.5 mM KH_2_PO_4_, 0.5 mM EGTA, 2 mM MgCl_2_, 5 mM pyruvate, 1 mM DTT) and 40 µM caspase-3 (Ac-DMQD-AMC) or caspase-8 (Ac-IEPD-AMC) fluorogenic substrate (Enzo Life Science GmbH, Lörrach, Germany). Fluorescence was measured every two minutes for two hours using a Tecan infinite 200 microplate reader (Tecan Group Ltd., Männedorf, Switzerland) and plotted over time. Caspase activity was then calculated from the slopes of the obtained curves.

### FADD recruitment, confocal laser scanning microscopy and image analysis

MF expressing the respective chimeric receptor (2×10^5^ cells) were seeded on 18 mm cover slips and cultured overnight. The following day, cells were transiently transfected with pEGFP-FADD plasmide using Lipofectamine™ 2000 (Invitrogen Life Technologies GmbH, Darmstadt, Germany) according to the manufacturer's instructions. 24 hours after transfection, cells were stimulated with antibody-crosslinked TRAIL (300 ng/ml sTRAIL crosslinked with 2 µg/ml anti-FLAG-M2 antibody (Sigma Aldrich), or 100 ng/ml Alexa Fluor 546-coupled recombinant human TNF. Subsequently, cells were washed (3×5 min, PBS), fixed (4% paraformaldehyde in PBS; 15 min at RT) and blocked (5% FCS, 0.05% Tween-20 in PBS, 30 min at RT). TRAIL stimulated cells were additionally immunostained with 1 µg/ml anti-mouse IgG Alexa Fluor® 546 (Molecular Probes, Invitrogen). Excess antibody and ligand was removed by washing with PBS, and coverslips were mounted on microscope slides using Fluoromount G™ (SouthernBiotech, Birmingham, USA), before being analyzed by confocal microscopy (Leica TCS confocal laser scanning microscope (Leica Mikrosysteme Vertrieb GmbH, Wetzlar, Germany) or Zeiss LSM710 microscope (Carl Zeiss MicroImaging GmbH, Jena, Germany)). Colocalization analysis was performed using the ImageJ [34] plug-in JACoP [35]. Pearson's correlation coefficient was calculated from optical sections of confocal z-stacks. Costes' method for automatic thresholding and testing of statistical significance of colocalization was applied [36]. Statistical analysis was performed using GraphPad Prism4.

### Flow cytometry and cell sorting

For flow cytometric analysis 2×10^5^ cells, for cell sorting 3×10^6^ cells were resuspended in PBA containing the primary antibody (anti-TNFR1 MAB225, 2.5 µg/ml; anti-TRAILR1 MAB347 or anti-TRAILR2 MAB6311, 4 µg/ml (all from R&D Systems Inc., Minneapolis, USA). After incubation for 1 hour on ice cells were washed in PBA and resuspended in PBA containing the secondary antibody (FITC-conjugated goat anti-mouse-IgG+IgM (H+L); Dianova) at a final concentration of 7.5 µg/ml. After 45 min of incubation cells were washed again and resuspended in 400 µl of PBA. Cells were analyzed using Beckman Coulter Cytomics FC 500, or sorted using Becton Dickinson FACS Vantage SE with *FACSDiVa*. For sorting, 3×10^4^ positive cells were collected in 5 ml of RPMI 1640+5% FCS containing 100 U/ml penicillin and 100 µg/ml streptomycin. Immediately after sorting cells were transferred into 6-well plates and cultured at 37°C and 5% CO_2_.

### Analysis of receptor internalization

2×10^5^ cells were seeded in 6-well plates and allowed to grow over night. The next day, plates were placed on ice, culture medium was removed and replaced with 1 ml RPMI+5% FCS containing 30 ng/ml ^125^I-TNF. To quantify nonspecific binding in addition a 200-fold excess of unlabeled TNF was present in the respective groups. After incubation on ice for 1 h plates were transferred to an incubator (37°C, 5% CO_2_) and incubated for different time periods. The plates were washed with phosphate-buffered saline (pH 7.0) followed either by an acid wash (50 mM glycine, 125 mM NaCl, pH 3.0) to disrupt accessible ligand-receptor interactions, or again with PBS. Cells were again washed twice with PBS, lysed by addition of 1 ml 1 M NaOH and radioactivity of the lysates was quantified using a γ-counter (LB 2100, Berthold, Bad Wildbad, Germany). Experiments were performed in duplicates. Radioactivity in the cell lysates of pH 3-treated groups corrected for unspecific binding was interpreted to represent internalized material, whereas the respective pH 7-treated group reveals cell surface bound plus internalized material.

## Supporting Information

Figure S1 **Cellular responses of MF-TRAILR1-Fas and MF-TRAILR2-Fas cells to TRAIL stimulation in absence of a secondary ligand crosslinkiner.** MF-TRAILR1-Fas cells (**A**) and MF-TRAILR2-Fas cells (**B**) were treated with serial dilutions of recombinant human FLAG-TRAIL (300–0.046 ng/ml; without crosslinking secondary antibodies) in presence (triangles) or absence (squares) of 0.5 µg/ml of the protein synthesis inhibitor cycloheximide or left untreated. Cell viability was determined by crystal violet staining after 18 h of stimulation. All experiments were performed in parallel: one representative experiment out of three is shown (mean values of triplicates ± SD). TRAILR1-Fas expressing cells show significant cytotoxic response only when co-stimulated with cycloheximide, whereas TRAILR2-Fas positive cells are responsive to TRAIL in the absence of additional stimuli.
(EPS)

Figure S2 **Kinetics of FADD-eGFP recruitment and cluster formation in MF-TRAILR1-Fas and MF-TRAILR2-Fas cells.** Colocalization of FADD-eGFP and Alexa Fluor 546-stained TRAIL was quantified using the ImageJ plug-in JACoP. Pearson's correlation coefficient (r_p_) was calculated from at least 15 cells per time point from three independent experiments (shown are mean values ± SD). For the unstimulated controls (t = 0 min) cells were incubated with FLAG-TRAIL (300 ng/ml, previously cross-linked with 2 µg/ml mouse anti-Flag M2 antibody, 1 h 37°C) for 15 min at 0°C to allow binding of the ligand under conditions of highly reduced membrane fluidity. Cells expressing TRAILR2-Fas (grey bars) show higher colocalization after 15 min stimulation (p<0.05, non-parametric Mann-Whitney U-test) compared to TRAILR1-Fas expressing MFs (white bars). Stimulation for 30 min or longer showed no significant difference, although the tendency for an enhanced FADD recruitment was clearly visible also after 30 and 60 min of stimulation in TRAILR2-Fas cells.
(EPS)

## References

[1] Aggarwal BB (2003) Signalling pathways of the TNF superfamily: a double-edged sword. Nat Rev Immunol 3: 745–756. 12949498 10.1038/nri1184

[2] LeBlanc HN, Ashkenazi A (2003) Apo2L/TRAIL and its death and decoy receptors. Cell Death Differ 10: 66–75. 12655296 10.1038/sj.cdd.4401187

[3] Emery JG, McDonnell P, Burke MB, Deen KC, Lyn S, et al (1998) Osteoprotegerin is a receptor for the cytotoxic ligand TRAIL. J Biol Chem 273: 14363–14367. 9603945 10.1074/jbc.273.23.14363

[4] Ashkenazi A (2002) Targeting death and decoy receptors of the tumour-necrosis factor superfamily. Nat Rev Cancer 2: 420–430. 12189384 10.1038/nrc821

[5] Fox NL, Humphreys R, Luster TA, Klein J, Gallant G (2010) Tumor Necrosis Factor-related apoptosis-inducing ligand (TRAIL) Receptor-1 and Receptor-2 agonists for cancer therapy. Expert Opin Biol Ther 10: 1–18. 19857186 10.1517/14712590903319656

[6] Gerspach J, Pfizenmaier K, Wajant H (2011) Therapeutic targeting of CD95 and the TRAIL death receptors. Recent Pat Anticancer Drug Discov 6: 294–310. 21762072 10.2174/157489211796957739

[7] Clancy L, Mruk K, Archer K, Woelfel M, Mongkolsapaya J, et al (2005) Preligand assembly domain-mediated ligand-independent association between TRAIL receptor 4 (TR4) and TR2 regulates TRAIL-induced apoptosis. Proc Natl Acad Sci U S A 102: 18099–18104. 16319225 10.1073/pnas.0507329102 PMC1312398

[8] Chan FK, Chun HJ, Zheng L, Siegel RM, Bui KL, et al (2000) A domain in TNF receptors that mediates ligand-independent receptor assembly and signaling. Science 288: 2351–2354. 10875917 10.1126/science.288.5475.2351

[9] Gonzalvez F, Ashkenazi A (2010) New insights into apoptosis signaling by Apo2L/TRAIL. Oncogene 29: 4752–4765. 20531300 10.1038/onc.2010.221

[10] Cha SS, Kim MS, Choi YH, Sung BJ, Shin NK, et al (1999) 2.8 A resolution crystal structure of human TRAIL, a cytokine with selective antitumor activity. Immunity 11: 253–261. 10485660 10.1016/s1074-7613(00)80100-4

[11] Hymowitz SG, Christinger HW, Fuh G, Ultsch M, O'Connell M, et al (1999) Triggering cell death: the crystal structure of Apo2L/TRAIL in a complex with death receptor 5. Mol Cell 4: 563–571. 10549288 10.1016/s1097-2765(00)80207-5

[12] Branschadel M, Aird A, Zappe A, Tietz C, Krippner-Heidenreich A, et al (2010) Dual function of cysteine rich domain (CRD) 1 of TNF receptor type 1: conformational stabilization of CRD2 and control of receptor responsiveness. Cell Signal 22: 404–414. 19879354 10.1016/j.cellsig.2009.10.011

[13] Mukai Y, Nakamura T, Yoshikawa M, Yoshioka Y, Tsunoda S, et al (2010) Solution of the structure of the TNF-TNFR2 complex. Sci Signal 3: ra83. 21081755 10.1126/scisignal.2000954

[14] Leverkus M, Sprick MR, Wachter T, Mengling T, Baumann B, et al (2003) Proteasome Inhibition Results in TRAIL Sensitization of Primary Keratinocytes by Removing the Resistance-Mediating Block of Effector Caspase Maturation. Mol Cell Biol 23: 777–790. 12529384 10.1128/MCB.23.3.777-790.2003 PMC140698

[15] Kelley RF, Totpal K, Lindstrom SH, Mathieu M, Billeci K, et al (2005) Receptor-selective mutants of apoptosis-inducing ligand 2/tumor necrosis factor-related apoptosis-inducing ligand reveal a greater contribution of death receptor (DR) 5 than DR4 to apoptosis signaling. J Biol Chem 280: 2205–2212. 15520016 10.1074/jbc.M410660200

[16] Lemke J, Noack A, Adam D, Tchikov V, Bertsch U, et al (2010) TRAIL signaling is mediated by DR4 in pancreatic tumor cells despite the expression of functional DR5. J Mol Med 88: 729–740. 20354842 10.1007/s00109-010-0619-0

[17] van Geelen CM, Pennarun B, Le PT, de Vries EG, De Jong S (2011) Modulation of TRAIL resistance in colon carcinoma cells: different contributions of DR4 and DR5. BMC Cancer 11: 39. 21272366 10.1186/1471-2407-11-39 PMC3045356

[18] Truneh A, Sharma S, Silverman C, Khandekar S, Reddy MP, et al (2000) Temperature Sensitive Differential Affinity of TRAIL for its Receptors: DR5 is the highest affinity Receptor. J Biol Chem 275: 23319–23325. 10770955 10.1074/jbc.M910438199

[19] Tur V, van der Sloot AM, Reis CR, Szegezdi E, Cool RH, et al (2008) DR4-selective tumor necrosis factor-related apoptosis-inducing ligand (TRAIL) variants obtained by structure-based design. J Biol Chem 283: 20560–20568. 18474604 10.1074/jbc.M800457200

[20] Reis CR, van Assen AH, Quax WJ, Cool RH (2011) Unraveling the binding mechanism of trivalent tumor necrosis factor ligands and their receptors. Mol Cell Proteomics 10: M110. 10.1074/mcp.M110.002808 PMC3013454 20852190

[21] Krippner-Heidenreich A, Tubing F, Bryde S, Willi S, Zimmermann G, et al (2002) Control of receptor-induced signaling complex formation by the kinetics of ligand/receptor interaction. J Biol Chem 277: 44155–44163. 12215450 10.1074/jbc.M207399200

[22] Rossin A, Derouet M, Abdel-Sater F, Hueber AO (2009) Palmitoylation of the TRAIL receptor DR4 confers an efficient TRAIL-induced cell death signaling. Biochem J 419: 185–192. 19090789 10.1042/BJ20081212

[23] Ouyang W, Yang C, Liu Y, Xiong J, Zhang J, et al (2011) Redistribution of DR4 and DR5 in lipid rafts accounts for the sensitivity to TRAIL in NSCLC cells. Int J Oncol 39: 1577–1586. 21769428 10.3892/ijo.2011.1129

[24] Feig C, Tchikov V, Schutze S, Peter ME (2007) Palmitoylation of CD95 facilitates formation of SDS-stable receptor aggregates that initiate apoptosis signaling. EMBO J 26: 221–231. 17159907 10.1038/sj.emboj.7601460 PMC1782382

[25] Lee HW, Lee SH, Lee HW, Ryu YW, Kwon MH, et al (2005) Homomeric and heteromeric interactions of the extracellular domains of death receptors and death decoy receptors. Biochem Biophys Res Commun 330: 1205–1212. 15823571 10.1016/j.bbrc.2005.03.101

[26] Micheau O, Tschopp J (2003) Induction of TNF receptor I-mediated apoptosis via two sequential signaling complexes. Cell 114: 181–190. 12887920 10.1016/s0092-8674(03)00521-x

[27] Schneider-Brachert W, Tchikov V, Neumeyer J, Jakob M, Winoto-Morbach S, et al (2004) Compartmentalization of TNF receptor 1 signaling: internalized TNF receptosomes as death signaling vesicles. Immunity 21: 415–428. 15357952 10.1016/j.immuni.2004.08.017

[28] Ashkenazi A, Pai RC, Fong S, Leung S, Lawrence DA, et al (1999) Safety and antitumor activity of recombinant soluble Apo2 ligand. J Clin Invest 104: 155–162. 10411544 10.1172/JCI6926 PMC408479

[29] Peter ME, Budd RC, Desbarats J, Hedrick SM, Hueber AO, et al (2007) The CD95 receptor: apoptosis revisited. Cell 129: 447–450. 17482535 10.1016/j.cell.2007.04.031

[30] Muhlenbeck F, Schneider P, Bodmer JL, Schwenzer R, Hauser A, et al (2000) The tumor necrosis factor-related apoptosis-inducing ligand receptors TRAIL-R1 and TRAIL-R2 have distinct cross-linking requirements for initiation of apoptosis and are non-redundant in JNK activation. J Biol Chem 275: 32208–32213. 10807904 10.1074/jbc.M000482200

[31] Wajant H, Moosmayer D, Wuest T, Bartke T, Gerlach E, et al (2001) Differential activation of TRAIL-R1 and -2 by soluble and membrane TRAIL allows selective surface antigen-directed activation of TRAIL-R2 by a soluble TRAIL derivative. Oncogene 20: 4101–4106. 11494138 10.1038/sj.onc.1204558

[32] Senes A, Engel DE, DeGrado WF (2004) Folding of helical membrane proteins: the role of polar, GxxxG-like and proline motifs. Curr Opin Struct Biol 14: 465–479. 15313242 10.1016/j.sbi.2004.07.007

[33] Huang DC, Cory S, Strasser A (1997) Bcl-2, Bcl-XL and adenovirus protein E1B19kD are functionally equivalent in their ability to inhibit cell death. Oncogene 14: 405–414. 9053837 10.1038/sj.onc.1200848

[34] Collins TJ (2007) ImageJ for microscopy. Biotechniques 43: 25–30. 10.2144/000112517 17936939

[35] Bolte S, Cordelieres FP (2006) A guided tour into subcellular colocalization analysis in light microscopy. J Microsc 224: 213–232. 17210054 10.1111/j.1365-2818.2006.01706.x

[36] Costes SV, Daelemans D, Cho EH, Dobbin Z, Pavlakis G, et al (2004) Automatic and quantitative measurement of protein-protein colocalization in live cells. Biophys J 86: 3993–4003. 15189895 10.1529/biophysj.103.038422 PMC1304300

